# Social Competence as a Positive Youth Development Construct: A Conceptual Review

**DOI:** 10.1100/2012/287472

**Published:** 2012-05-02

**Authors:** Hing Keung Ma

**Affiliations:** Department of Education Studies, Hong Kong Baptist University, Hong Kong

## Abstract

Social competence is defined in terms of interpersonal relationships, self and group identities, and development of citizenship. While the focus of the author's previous research is on relationship and identity, the main focus of this paper is on the development of citizenship. A 4-stage developmental model of citizenship is proposed. A brief discussion of the educational implication of each of the stages is presented. The issues concerning the assessment of social competence are clearly delineated, and the discussion serves as a basis for future studies. Finally, five current issues concerning the launch of the “Moral and National Education (MNE) Subject” in Hong Kong primary and secondary schools are discussed.

## 1. Background

Our educational goal is to foster the development of a clear national identity and citizenship in Hong Kong adolescents. Recently, the government of the Hong Kong Special Administrative Region (HKSAR) proposed to launch the Moral and National Education (abbreviated as MNE hereafter) Subject in the primary education sector in September 2012 and then in the secondary education sector in September 2013. This subject is an independent subject to be taught in Grade 1 through 12 (i.e., P1 to P.6 and S1 to S6), and it takes one to two class periods a week (one class period is usually 35 to 40 minutes long in most schools). The government has produced a curriculum guide and has conducted an open consultation to the public, which had just finished on 31 August 2011. The debate on whether we should have an independent subject focusing on national education has received considerable criticism and resistance from the teachers, secondary school students, and politicians. In this paper, we will delineate the concept of social competence with special reference to national identity and citizenship. We believe that a good understanding of the concept of social competence will be useful for the implementation of civic education, citizenship education, national education, whole-person education, and positive youth development education. We will also discuss the current issues related to the teaching of social competence. Some of the issues are related to the implementation of the MNE subject in schools.

## 2. Definition of Social Competence

There are three important aspects of social competence, which refers to (a) the ability to build positive and healthy interpersonal relationships and to resolve interpersonal conflicts, (b) the development of a clear self-identity in general, and a group or collective identity (e.g., national identity) in particular, and (c) the orientation to be a responsible citizen in one's society and a caring citizen in the world. The development of a positive and supportive relationship with different types of people (e.g., parents, siblings, relatives, peers, teachers, and other adults) is an important adaptation skill that children and adolescents must acquire in order to live happily in our society. In particular, adolescents should be taught to understand the possible conflicts with others and to learn a set of interpersonal negotiation strategies for use with peers and adults. Another important task is the development of a clear identity, especially a national identity. The concept of identity includes a complete concept of self, which consists of personal identity, group identity, social identity, national or racial identity, vocational or occupational identity, sex-role stereotype, religious identity, and so on. The development of a firm and coherent sense of where you fit into your society and country is regarded as a significant aspect of identity and psychosocial development of an adolescent. The discussion on human or interpersonal relationships and the search for identity have been thoroughly discussed in Ma [1]. The present paper will focus on the development of citizenship and its related issues.

## 3. A Developmental Model of Citizenship

Ma [2] has proposed an integrated model of moral development and moral education based on a number of contemporary psychological theories of moral development. There are four parameters in the model: (a) Human Relationship and Altruism, (b) Moral Feeling and Psychological Needs, (c) Moral Judgment, and (d) Citizenship. The following 4-stage developmental model of citizenship is based on the fourth parameter in Ma's [2] theoretical model.

### 3.1. Stage 1: Discipline and Compliance

According to Erikson's theory of psychosocial development, young children at this developmental stage develop tendencies to explore and experiment their initiatives in family and school settings. Constant discouragements from the adults on their initiatives will lead to a feeling of guilt. Older children at this level tend to develop a sense of industry in their learning. Constant failure in their learning will lead to the development of a sense of inferiority [3, pages 53–56]. In other words, children at this level will develop a clear social self in the interaction with others in family and school settings. One thing that they should learn about is the discipline and rules in a group.

Young children at this level tend to be egocentric in their social perspective taking. They are unable to think and reason from other's perspective. They think that what they see and feel is what all the others see and feel.

One main reason for young children to obey rules and regulation at this level is to avoid punishment, especially physical punishment. Rules and regulations are usually set by adults (e.g., parents or teachers). For older children, the compliance to social norms, propriety, and common practices is based on instrumental purpose and equal exchange. For example, keeping a promise is a kind of propriety or a norm in a society. But one would think that it is right not to keep a promise to a person who has not kept a promise to him or her in the past. If anyone does not comply with the norm, people at this stage would think that it is right to revenge or retaliate.

Children must learn to discipline themselves and to have respect for and comply with group rules at this level. Durkheim [4] has proposed that there are three important elements of morality: discipline, group affiliation, and autonomy. The discipline element is the major concern of this developmental stage, while the other two elements are focused at the next stage.

### 3.2. Stage 2: Belongingness and Group Affiliation

The development of a clear group identity and the respect of the social rules and regulations within the group are essential for building a positive relationship between an individual and a group (e.g., the class or school house one belongs to). People at this stage would live up to the group's expectations. The group refers in general to primary groups (e.g., family, class, school house, or religious group). The general expectation is that one should be good and nice to all in-group members and tries his or her best to bring honor and benefits to the group members. The main task at this developmental stage is the development of a positive identity and relationship with one's family. The development of a sense of belonging to one's family forms a basis for the development of an advanced level of social competence. Research has indicated that in the socialization process, family has a large impact on children's developmental outcomes [5]. Ma and his colleagues [6] found that Chinese adolescents with good relationships with their parents showed lower frequency of antisocial behaviors than did Chinese adolescents with bad relationships. In another study, his data indicated that altruistic orientation in Chinese children was directly associated with a positive family social environment [7].

 Children at this stage are able to put their feeling in other's perspective. They are more willing to think from another's role in their social interaction. They are not only able to put one's foot in other's shoes, but they can also think and argue from a third-person perspective and group perspective.

 At Stage 1, the educational emphasis is on discipline by authority (e.g., parents and teachers); however, the educational emphasis at Stage 2 is more on group identity and self-discipline, an autonomous, independent, and free decision on complying to the group rules and regulations because you treasure the identity of the group. 

### 3.3. Stage 3: Social Conscience and National Identity

One of the goals of this level is the “development of roles and skills that will prepare adolescents to take a meaningful place in adult society” [3, page 54]. Toward the end of this developmental stage, adolescents should have developed a comprehensive social identity including gender-role identity, vocational identity, religious identity, and national identity. People at this stage should develop a social conscience or an orientation to care for others, in particular, the old, the very young, and the weak in one's country for the sake of maintaining the dignity of life. They should have an affective concern for others' welfare and rights. Many adolescents at this level are willing to help others not because of social exchange and reciprocity but because of a personal willingness or obligation and a deep respect for life. The care for people in one's country and the love of one's country including its history, culture, tradition, and values is an important basis of national identity. This is a law-abiding and law-maintaining stage. People would strive for the prosperity and stability of one's nation rationally and legitimately.

People at this stage are able to think and argue from a society's or from one's country's perspective. The welfare of one's community and one's nation is the major concern in one's social interaction with others. Hall [8, page 4] argued that “the social construction of nationhood is inevitably carried out via ‘the marking of difference and exclusion.' This is often especially prevalent within history teaching.”

While the development of national identity and the respect for the social and political institutions is the major focus of education at this stage, we should emphasize the value of peace and friendship in building up the relationship with other countries.

### 3.4. Stage 4: Global Perspectives and World Citizenship

In contrast to the development of national identity in Stage 3, the emphasis here is to break the boundary between countries and to foster the development of world citizenship at this stage of citizenship.

According to Cogan [9], there are five attributes of citizenship. We will try to apply these five attributes in the elaboration of world citizenship here. (a) A sense of global identity: students should be taught not only the concept of national identity and national patriotism but also multinational identity and world citizenship. Not only do they belong to their own country but they also belong to this world. They should show care and empathy to those disadvantaged people in the less developed places in the world and thus should try to help them as far as possible. (b) Human rights and entitlements: people in the world should have the essential basic rights such as right to life, right to seeking happiness, right to freedom of speech, and right to properties (c) General responsibilities, obligations, and duties: as a responsible world citizen, one should have the obligation to obey international law and to respect the rights, traditions, and customs of other people in the world. (d) Active in public and international affairs: a good world citizen participates actively in public and international affairs and shows concern and care for the welfare of the people in the world. (e) Acceptance of basic societal values: there are some essential and fairly universal societal values that everyone should accept. For example, “trust, cooperation, respect for human rights, non-violence, and so on” [9, page 5].

People at this stage tend to think and reason from a multinational or global perspective. They are more concerned with the welfare of all the people in the world. They will develop a multinational identity or world citizenship that places emphasis on basic human rights and universal societal values. The development of the genuine world citizenship should be the aim of citizenship education. To be a world citizen does not mean that one would forsake one's nation. Instead, one should love all the other countries of the world as though they are the same as your own country. 

## 4. Assessment of Citizenship

There are three main issues in the assessment of citizenship [10]. The first issue is the purpose of assessment. Do we regard it as a formative assessment or a summative assessment? How frequent will the assessment take place? Since the teaching of citizenship is not regarded as something similar to subject teaching such as language, mathematics, science, and history, the purpose of assessment of citizenship is not necessarily knowledge-based or academically oriented. As such, formative assessment at a less frequent level, for example, once every term or every quarter of an academic year is more desirable. The contents of formative assessment should include the following: (a) prosocial behavior: how frequent of prosocial behavior (e.g., doing voluntary work and getting an award in external competition) the student displays in the past term? (b) Antisocial behavior: how frequent of antisocial or negative behavior (e.g., late to school, late in submission of homework, and fighting with classmates) the student displays in the past term? (c) Social and communication skills: is the student able to interact and communicate with others effectively and gracefully? For example, is the student able to say sorry to others sincerely after his/her wrongdoings? Is the student able to accept others' apologies gracefully? Is the student able to listen to other's opinion, advice, or criticism humbly? The second issue is what sort of evidence or data do we need to collect for the purpose of assessment? The evidence should not be limited to knowledge but should include a comprehensive range of evidences such as group discussion, project-based learning, practical experiences, voluntary work, field studies, and research exploration. The format of assessment should also not be limited to class work such as quizzes, class discussion, and group presentation but also include, for example, direct observation of the students' behavior, peer nomination or peer reputation, the use of peer rating scales, exhibition of project products, and sharing of social and voluntary work. The third issue is on assessment outcome. What kind of assessment outcome should it take? Should we give a grade to each of the students and should we record this result as part of his or her academic performance in one's annual assessment report? It is suggested that a description of the achievement, learning attitude, strengths to be maintained, and weaknesses to be improved should be provided to each student on a separate report. The assessment of citizenship is very complicated. Reliable and valid methods have yet to be developed; it is perhaps better to take the assessment in the form of formative one. The teacher should give feedback based on learning outcomes to the students and their parents in the form of qualitative and descriptive remarks. 

## 5. Antecedents of Social Competence

In their studies in Hong Kong, Ma and his colleagues found that Chinese adolescents' good relationships with parents, peers, and teachers were negatively associated with their antisocial or delinquent behaviors [6, 11]. In other words, good interpersonal relationships tend to reduce the frequency of delinquent behaviors in adolescents. In addition, Robert Sternberg emphasizes the role of environmental context on intelligence, and he argued that “intelligent behavior involves adaptation” to environment [12, page 203]. He also suggested that social competence is a general and adaptive skill in Western culture. In general, a positive social environment with good social supports favors that development of social competence.

Harter [13] in an intensive review of the concept of self concluded that the self served as antecedents of cognitive and social construction in children. It is common to classify the self into two categories: I-self (i.e., self as subject) and Me-self (self as object). McDevitt and Ormrod [12] suggested a few ways to enhance the sense of self of students. Two of the strategies are (a) To “promote success on academic, social and physical tasks” (page 454): teachers and parents should give challenging tasks and assignments to students and should provide support and guidance for them to achieve. (2) To “focus students' attention on their improvement rather than on how others are performing” (page 456): in the development of positive self-concepts, it is more productive and constructive to help students to make their own progress and improvement instead of comparing their achievements with the peers.

In summary, both the social environment (whether the relationship with parents, peers, teachers, and others is good or not, and whether the social supports provided by these people are adequate or not) and the self (whether the concept of self is positive, stable, and clear or not) are essential antecedents of social competence.

## 6. Relationship between Social Competence and Adolescent Developmental Outcomes

Prosocial behavior is frequently used as a measure of social competence [14]. Adolescents with a high level of competence exhibit high frequency of prosocial behavior. In addition, social competence is positively associated with academic achievement. Wentzel [15] found that children who had high peer status and displayed prosocial and responsible behaviors are usually high academic achievers. In a study of 2,862 Hong Kong Chinese adolescents, Ma et al. [11] also found that prosocial behavior tended to have a positive relationship with academic achievement.

Social competence appears to be associated with emotional competence, especially in girls [16]. Social skills and social effectiveness are positively related to emotion knowledge in children. According to a review by Saarni and her colleagues, they concluded that “children who demonstrated more complex emotion understanding were more accepted by their peers” [16, page 257].

 Erikson [17] proposed that the search for identity is the main developmental task that adolescents have to face. The achievement of a clear identity contributes to the development of self. Failure to achieve a clear identity will lead to role confusion. Marcia [18] classified the adolescents in four different identity statuses: achievement, moratorium, foreclosure, and diffusion. A detailed description of these identity statuses is presented in [1]. In the study of the characteristics of the four identity statuses of Hong Kong Chinese adolescents in terms of prosocial and delinquent behaviors, Ma et al. [6, page 76] found the following. (a) Adolescents in the Identity Achievement group tended to display high frequency of prosocial behavior and low frequency of delinquent behavior. (b) Adolescents in the Identity Moratorium group usually exhibited high frequencies of both prosocial and antisocial behaviors. (c) Adolescents in the Identity Foreclosure group exhibited fairly high frequencies of prosocial behaviors and low frequencies of antisocial behaviors. (d) Adolescents in the Identity Diffusion group exhibited low frequencies of prosocial behaviors and high frequencies of antisocial behaviors. In some sense, the social behavior pattern of the adolescents in the Identity Achievement group is the most promising and fulfils the social expectations of parents and teachers, whereas the social behavior pattern in the Identity Diffusion group is the least acceptable by the parents and teachers.

## 7. Practical Ways to Promote Social Competence in Adolescents

The practical ways to promote social competence in adolescents are similar to those to promote moral competence [19] and include the following.

- School-based teaching packages should not only focus on social competence but also include the other positive youth development constructs in their curriculum development. In other words, a comprehensive teaching package dealing with the all-round or whole-person development is more effective.

(b) Parent education is essential. Parents should be taught to understand the concepts of social competence and the techniques in fostering the development of social competence in adolescents.

(c) A positive learning environment with a lot of social supports from parents, teachers, peers, and the general public should be established. The environment should emphasize on caring for others, social justice, respect, and responsibility. In addition, the school environment should be peaceful and nonthreatening for open and free discussion, exchange of ideas, sharing of feelings and experiences, practicing democracy, and for social action including voluntary social services.

## 8. Current Issues on the Moral and National Education (MNE) Subject

As mentioned at the beginning of this paper, the government of the Hong Kong Special Administrative Region (HKSAR) proposes to launch the Moral and National Education (MNE) Subject in primary education sector in September 2012 and then in the secondary education sector in September 2013. The proposal has generated a lot of hot debates throughout society. I have chosen five issues to be discussed here because of their general social and educational implications.

**Brainwashing Issue**

Some people criticized the possible brainwashing nature of the MNE subject. That is, the subject attempts to indoctrinate national patriotism and to elicit affection from students towards the political leaders and the unilateral appreciation of the recent economic, scientific, and sports achievements in China. First of all, to love one's country does not mean that one has to love everything in one's country. One can be critical of the political strategy, the administrative policy, and the public behavior of the political leaders of one's country because of one's intention to help the country to progress.

One effective way to prevent brainwashing in education is to foster the development of autonomy in students. Many psychologists and educators [20–22] regard personal autonomy as an important characteristic of the highest stage of moral judgment. Bull [20] argues that personal autonomy “alone is wholly adequate in a democratic and increasingly permissive society. It follows that autonomy must be the true goal of moral education” (page 121). According to Bull [20], there are three enemies of personal autonomy: (a) authoritarianism: teachers should not regard themselves as a powerful authority in the delineation of the concepts of morality and national issues. Instead, they should take an open and objective attitude in their teaching. A nonthreatening and peaceful classroom or school social environment favors free and constructive interaction between the students and teachers. (b) Physical discipline: physical discipline is not allowed in Hong Kong schools and should not be practiced in family settings. Physical discipline is not only an enemy of autonomy but is also associated with delinquency and child aggression [23]. (c) Indoctrination: intensive indoctrination in the teaching of morality and national issues is harmful to the development of autonomy and creativity. Students should be given ample opportunities to study and investigate a topic from different perspectives and by using different methods. After all, they should have the autonomy and freedom to choose their own values and beliefs.

**Assessment Problem**

The assessment of morality, national identity, social competence, or citizenship is very difficult and complicated. The method of assessment tends to be less reliable and valid [22]. In view of the technical inadequacy in the assessment, it is suggested that the assessment of the MNE subject should be formative rather than summative. Teachers should compare the progress of the student in this MNE subject with the student himself or herself and not with other students. Descriptive remarks on knowledge acquisition, social skills, and social attitudes should be provided. Chinese parents tend to place great emphasis on proper conduct, moral behavior, and academic achievements in the socialization of their children [24, 25]. If the MNE subject does not involve a public examination or school grade, then it will be more acceptable to parents at this stage. In addition, the less formal and less high-staking nature of this subject in comparison to other traditional subjects (e.g., language, mathematics, science, or geography) will provide some valuable opportunities for both teachers and students to teach and learn this subject in a less stressful and more autonomous manner.

**Individual Subject versus Hidden Curriculum**

Some people argue that the teaching of morality and social competence including national issues happens in most of the subjects (e.g., language, history, economic and public affairs, and liberal studies) and in most educational occasions (class teacher lessons, assembly, outside class activities, field trips to China, etc.). In some sense, MNE is practiced as a hidden curriculum in all schools. Then, why is it necessary to treat it as an independent and required subject in all primary and secondary schools? Unlike the teaching of intellectual subjects such as physics, chemistry, mathematics, and history which have clear syllabus or subject contents, the task of moral education is less defined and less explicit. Duncan [26] argues that because of this unclear understanding of the task of moral education, “the greatest temptation to seek an escape from moral thinking arises in the field of moral education” (page 15). Teachers of subjects such as literature, history, integrated science, and economic and public affairs that unavoidably involve some important moral issues often receive no proper and systematic training in moral thinking. The same argument applies to national education. The setting up of MNE as an independent subject is the first step to build up a structure for the curriculum development, teacher training, and systematic investigation of teaching methods in moral and national education.

**Teacher Training**

As we just pointed out, one obvious advantage in the setting up of MNE as an independent subject is perhaps on formalizing the teacher training in the field of moral, national, social, and citizenship education. Howard Gardner [27] has proposed a theory of multiple intelligences, Catalano et al. [28] has established a theory of 15 positive youth development constructs, and Ma [2] has also put forward a theory of whole-person development for school education. The three theories put emphasis not only on cognitive competence but also on social and moral competences. The launch of MNE subject will open a new page in teacher education. Teachers of the MNE subjects will have opportunities to learn the theories of moral and social development at an advanced level and also to learn and explore the various methods in the teaching of the MNE subject.

**National Identity versus World Citizenship**

Some critics of the MNE subject argued that we should not be too narrow-scoped in our teaching of the next generation: we should teach them to be a world citizen instead of a Chinese citizen. The increasing heated debates on the conceptions of Chinese national identity and its suspected discrepancies with the conceptions of global or world citizenship identity in public discourse are meaningful and inspiring, though could be somewhat confusing and overwhelming to our children and adolescents. According to our developmental model of citizenship mentioned above, people develop the concept of national identity before they develop the concept of world citizenship. In addition, the concept of world citizenship includes the following orientation: one should love all the other countries of the world as though they are the same as your own country. It is argued that the skipping of the third stage is usually unlikely to happen in common children. The developing adolescents require guidance and help from their parents and teachers in order to equip them with the critical capacity to sensibly evaluate alternative and sometimes contradictory national narratives within Hong Kong society to enhance a healthy development of a desirable national adhesion, a prudent share of national values, and eventually and hopefully the value and concept of world citizenship.

## 9. Conclusive Remarks and Suggestions for Future Research

Social competence is defined in terms of interpersonal relationships, self and group identities, and development of citizenship. While the focus of Ma's [1] previous paper is on relationships and identities, the main focus of this paper is on the development of citizenship. A 4-stage developmental model of citizenship is proposed. While the theoretical model is constructed based on contemporary psychological theories, the stage pattern should be empirically tested in future studies. The developmental sequence of the four stages should be investigated by longitudinal studies. In addition, cross-cultural data from other countries including Western countries would be useful in testing the cultural universality of the stages. A brief discussion of the educational implication of each of the stages is presented. A more detailed investigation of the implication of each stage for teaching and learning will be useful for the construction of teaching materials on citizenship.

 The issues concerning the assessment of social competence are clearly delineated. The discussion serves as a basis for future studies. For example, what strategy is there that will make the assessment of social competence more reliable and valid?

 Finally, five current issues concerning the launch of the “Moral and National Education (MNE) Subject” in Hong Kong primary and secondary schools are discussed. While the proposed MNE subject appears to have some educational merits, the successful implementation of this subject depends on the following factors: (a) the government must seek general support from all the stake-holders (parents, students, politicians, and the public) of concern. (b) A more systematic and structural reorganization of all the related subjects (e.g., liberal studies, integrated humanities, life and society) has to be conducted to avoid overlapping. (c) Formal teacher education courses focusing on the teaching of the MNE subject are to be included in the B.Ed. degree course as well as the Postgraduate Diploma in Education course. (d) Research (especially long-term and intensive studies) on all aspects of moral and national education is to be encouraged and supported by the government. (e) A more flexible and realistic implementation schedule must be considered. For example, is it possible to launch this MNE subject in all primary schools in September 2012, then in all Form One (Grade 7) classes in September 2013, finally in all Form 4 (Grade 10) classes in September 2016? If everything goes fine, this subject will be taught in all primary and secondary schools (Grade 1 to Grade 12) starting from September 2018.
